# On the coordination of highly dynamic human movements: an extension of the Uncontrolled Manifold approach applied to precision jump in parkour

**DOI:** 10.1038/s41598-018-30681-6

**Published:** 2018-08-15

**Authors:** Galo Maldonado, François Bailly, Philippe Souères, Bruno Watier

**Affiliations:** 1 LAAS-CNRS, Université de Toulouse, CNRS, UPS, Toulouse, France; 2 LAAS-CNRS, Université de Toulouse, CNRS, Toulouse, France

## Abstract

The human body generally has more degrees of freedom than necessary for generating a given movement. According to the motor abundance principle, this redundancy is beneficial as it provides the central nervous system with flexibility and robustness for the generation of movements. Under the hypothesis of the Uncontrolled Manifold, the additional degrees of freedom are used to produce motor solutions by reducing the variability that affects the motion performance across repetitions. In this paper, we present a general mathematical framework derived from robotics to formulate kinematic and dynamic tasks in human movement. On this basis, an extension of the Uncontrolled Manifold approach is introduced to deal with dynamic movements. This extension allows us to present a complex experimental application of the proposed framework to highly dynamic task variables in parkour movements. This experiment involves dynamic tasks expressed in terms of linear and angular momenta. The results show that the central nervous system is able to coordinate such skilled tasks which appear to be preferentially controlled and hierarchically organized. The proposed extension is promising for the study of motion generation in anthropomorphic systems and provides a formal description to investigate kinematics and dynamics tasks in human motions.

## Introduction

Motor redundancy^1^ provides the human body with more degrees of freedom than necessary to achieve a given motor task. It requires the central nervous system (CNS) to synthesize a family of control solutions that guarantee the execution of a given task. Different approaches have been proposed to explain how the CNS solves motor redundancy, by means of degrees of freedom reduction^2^, optimization^3^, or optimal feedback control^4^. Motor redundancy can be viewed as a benefit rather than a problem for the neuromotor system (motor abundance principle^5^). According to the motor abundance principle, redundant degrees of freedom are used to better achieve a steady motor solution in the sense of being robust against perturbations (internal and external ones). Given the context, the movement might have to cope with several tasks at the same time (e.g. when trying to open a door while holding a coffee cup, at least two tasks are likely to be preferentially controlled: turning the handle and not spilling the coffee). To deal with this apparent conflict, it has been proposed that the human brain could organize tasks hierarchically to generate the motion^6,7^.

The uncontrolled manifold (UCM) approach^8^ is based on the hypothesis that movement organization can be described in terms of motor tasks being preferentially controlled by the CNS. In this context, the stability of the motor solution is evaluated in terms of inter-trial motor variability across repetitions. The variability of elemental variables from a reference behavior (e.g. the mean joints configuration) that alters the nominal task achievement, can be expressed using the task Jacobian which maps the space of elemental variables (e.g. joints configuration) to the motor task space. This comes from the property of the null space of the task Jacobian, in which elemental variables variations do not affect the task execution, while any variation in its orthogonal space affects it. This projected space is called UCM. The UCM approach states that when the variance of elemental variables in the UCM (identified as good variability) is greater than the variability onto its orthogonal space (identified as bad variability), then the motor task is considered as being preferentially controlled by the CNS. The good variability can be interpreted as a motor synergy in which co-varying elemental variables contribute to the stability of a motor task.

The UCM approach has been applied to the study of various motor tasks. Postural tasks have been studied through muscles modes^9^ and joint coordination^10^. Tasks such as pointing^11^, reaching and grasping^12^, sit-to-stand^13,14^, and drawing^15^ have also been studied through joint coordination. Force production tasks have been investigated by considering finger forces^16^ and joint torques^17^. However, most of these studies only accounted for quasi-static motor tasks, although most of human movements are inherently dynamic. In the study of Yen *et al*.^17^, the UCM approach was applied to study force tasks in single leg hopping using the dynamic consistent Jacobian^18^. This mathematical formulation can only be applied for mono-contact force production tasks. Furthermore, this study considered segment angles as elemental variables in spite that sensory receptors provide information to the CNS about changes in joint angles according to physiology. In fact, joint segments are dependent variables and thus changing a segment angle will modify other segment angles in the kinematic chain. The UCM approach has also been applied to study linear and angular momenta expressed at the CoM^13^ in a sit-to-stand task and the angular momentum in human walking^19^. The later study was preceded by a principal component analysis (PCA). This implies dimensionality reduction into principal components whose origin is not well understood and which contradicts the motor abundance principle.

In this paper, we propose a general framework to extend the formalism of the UCM approach^8^ to tasks in which movement dynamics matter. This framework requires to consider higher order derivatives of the joint variables and respects the motor abundance principle. The first contribution of this paper is to propose an extension of the UCM approach for studying the organization of movements which are inherently dynamic. To this end, we present a general formulation inspired from the formalism of poly-articulated systems and the task function approach developed by roboticists^20,21^ that permits to express kinematic and dynamic motor tasks. Then, we present an experimental validation applied to specific whole-body parkour motions in which we demonstrate that the CNS is able to coordinate degrees of freedom to achieve such an inherent highly dynamic and skilled task. We also show a time-dependent hierarchical organization of tasks.

## Mathematical Background and Contributions

### Task function formalism in robotics and the UCM approach

The task function approach in robotics^20,21^ can be used to express motor task variables of anthropomorphic systems motion. A configuration ${\bf{q}}\in {\mathscr{Q}}$ of the body is defined by *k* + 6 parameters, where the first 6 variables describe the pose (position and orientation) of a reference frame (usually called root frame) with respect to the world frame and the *k*–remaining variables represent the angular value of joints. ${\mathscr{Q}}$ is a *n* = *k* + 6–dimensional manifold which can be locally identified to ${{\mathbb{R}}}^{n}$^21^. In order to express human behaviors at the kinematic level, motor tasks can be expressed in terms of positioning specific body segments. Let us denote by ${\bf{q}}(t)\in {\mathscr{Q}}$ a configuration of the body at time *t*. For notational convenience the explicit dependence on *t*, will be omitted except when necessary. Let ${\mathcal M}$ be the task space and let us denote by ${\bf{e}}({\bf{q}})\in  {\mathcal M}$ a task function. ${\mathcal M}$ is a *m*− dimensional manifold which can be locally identified to ${{\mathbb{R}}}^{m}$^21^. A task function comes down to an output error function whose regulation to zero corresponds to the execution of the task. For instance, a pointing task can be defined by the task function **e**(**q**) = **hand**(**q**) − **hand**_*target*_, which describes the gap between the current hand position **hand**(**q**), when the body is at configuration ***q*** and the targeted hand position **hand**_*target*_. In order to express the variation of the task function with respect to the body configuration, the task Jacobian ${J}_{e}({\bf{q}})=\frac{d{\bf{e}}({\bf{q}})}{d{\bf{q}}}$ is used. *J*_*e*_(**q**) is a *m* by *n* matrix whose entries are defined as:

1 $$\forall i\in \mathrm{1,}\,...,\,m,\,\forall j\in \mathrm{1,}\,...,\,n,\,{J}_{e}{({\bf{q}})}_{ij}=\frac{\partial {e}_{i}}{\partial {q}_{j}}.$$

Based on this formulation, the UCM approach^8^ can be easily formulated as follows. Consider that *N*_*r*_ repetitions of a studied movement have been recorded with one participant. For *r* = 1, ..., *N*_*r*_, let us define by ***q***_*r*_(*t*) and ***e***(***q***_*r*_(*t*)) the joint configuration trajectory and the corresponding task function trajectory for the *r*^*th*^ repetition respectively. Let $\bar{{\bf{q}}}(t)$ denote the mean value of the joints configuration trajectory across the *N*_*r*_ repetitions. At each time *t*, given the definition of the task Jacobian, the first order Taylor expansion of a task **e**(**q**) in the neighborhood of $\bar{{\bf{q}}}$ yields:

2 $$\forall r=\mathrm{1,}\,\mathrm{...},\,{N}_{r},\,{\bf{e}}({{\bf{q}}}_{r})-{\bf{e}}(\bar{{\bf{q}}})={J}_{e}(\bar{{\bf{q}}})({{\bf{q}}}_{r}-\bar{{\bf{q}}})+{\boldsymbol{\varepsilon }}({{\bf{q}}}_{r}),$$

where $\varepsilon ({{\bf{q}}}_{r})$ contains higher order terms of the expansion known as residual.

Considering the residual as negligible, for each repetition *r*, one can compute the projection ***ucm***_*r*_ and **ucm**_⊥,*r*_ of the joint configuration deviation ${{\bf{q}}}_{r}-\bar{{\bf{q}}}$ respectively onto the null space of the Jacobian matrix ${J}_{e}(\bar{{\bf{q}}})$ and onto its orthogonal space at each time as follows:

3a $${\bf{uc}}{{\bf{m}}}_{r}={P}_{e}({{\bf{q}}}_{r}-\bar{{\bf{q}}}),$$

3b $${\bf{uc}}{{\bf{m}}}_{\perp ,r}=({{\bf{q}}}_{r}-\bar{{\bf{q}}})-{\bf{uc}}{{\bf{m}}}_{r},$$

where ${P}_{e}=1-{J}_{e}^{\#}{J}_{e}$ is the projector onto the null space of *J*_*e*_ (e.g. *P*_*e*_*J*_*e*_ = 0 and *P*_*e*_*P*_*e*_ = *P*_*e*_). The inter-trial variance of ${\bf{uc}}{{\bf{m}}}_{r=1,\mathrm{...},{N}_{r}}$ and ${\bf{uc}}{{\bf{m}}}_{\perp ,r=1,\mathrm{...},{N}_{r}}$ respectively denoted by ${V}_{ucm}\in {\mathbb{R}}$ and ${V}_{uc{m}_{\perp }}\in {\mathbb{R}}$, are then normalized by the dimension of each space with ${N}_{ucm}=dim({\mathscr{Q}})-dim( {\mathcal M} )$ and ${N}_{uc{m}_{\perp }}=dim( {\mathcal M} )$. Note that in order to report the variability of **ucm**_*r*_ and **ucm**_⊥,*r*_ independently, one should also normalize by the number of repetitions. Then, an index of motor task control “ITC” can be calculated as the ratio between both variances:

4 $${V}_{ucm}=\sum _{r=1}^{{N}_{r}}\frac{1}{n}||{\bf{uc}}{{\bf{m}}}_{r}{||}_{2}^{2},$$

5 $${V}_{uc{m}_{\perp }}=\sum _{r=1}^{{N}_{r}}\frac{1}{n}||{\bf{uc}}{{\bf{m}}}_{\perp ,r}{||}_{2}^{2},$$

6 $$ITC=\,\mathrm{ln}(\frac{{V}_{ucm}\cdot {N}_{uc{m}_{\perp }}}{{V}_{uc{m}_{\perp }}\cdot {N}_{ucm}}).$$

According to the UCM hypothesis, when the ITC is greater than 0, the motor task is considered as being preferentially controlled by the CNS^8,22^. Furthermore, the ITC can be used to quantify a hierarchical structure of motor tasks according to their apparent importance in the generation of the motion. Note that when applying the UCM approach, the task function is defined as a vector of specific variables that are hypothesized to be preferentially controlled by the CNS. Through the suitable task Jacobian, these variables are related to the subset of configurations that depend on the considered motion. For example, in a pointing task, the researcher might consider only the finger position (dim(**e**(**q**)) = 3) and the upper-limb joints.

In its classical formulation the UCM approach proposes to deduce from the analysis of variability structure which tasks are preferentially controlled by the CNS. To this end, the considered task function **e**_r_(***q***) is expressed as joint position error between the current trial configuration of the joints and a mean reference behavior. In order to extend this approach to the study of the coordination of tasks where movement dynamics matters, we propose to consider candidate task functions that depend on higher order derivatives of the elemental variables. In the next section, we introduce the generalized framework for expressing these motor tasks.

### Extending the UCM approach to dynamic tasks

The task Jacobian (Eq. (1)) is known to be the linear mapping between the time derivative of the task and the velocity of the elemental variables:

7 $$\dot{{\bf{e}}}({\bf{q}},\dot{{\bf{q}}})={J}_{e}({\bf{q}})\dot{{\bf{q}}}.$$

In turn, $\dot{{\bf{e}}}({\bf{q}},\dot{{\bf{q}}})$ can be viewed as a new task function depending on **q** but also on $\dot{{\bf{q}}}$. This task function is well suited for describing differential behaviors as speed tracking tasks for instance. This representation of motion is still decoupled from the dynamics of the system and thus it does not take into account inertial effects that are essential for analyzing the movement dynamics. Now, if one wants to express the motion at the level of the dynamics, then task functions need to be related to higher order derivatives of the configuration variables. Indeed, it is well-known that rigid body dynamics is expressed as a function of the second order derivative of the joints variables^23^. In order to express the system dynamics in the task space, Eq. (7) must be time differentiated:

8 $$\ddot{{\bf{e}}}({\bf{q}},\dot{{\bf{q}}},\ddot{{\bf{q}}})={\dot{J}}_{e}({\bf{q}},\dot{{\bf{q}}})\dot{{\bf{q}}}+{J}_{e}({\bf{q}})\ddot{{\bf{q}}},$$

where $\dot{J}({\bf{q}},\dot{{\bf{q}}})\dot{{\bf{q}}}$ can be viewed as a dynamic drift of the task corresponding to nonlinear effects. In robotics, this expression has been used to formulate different kind of tasks, for instance tasks for visual servoing^24^ or center of mass (CoM) trajectory control^25^.

In the sequel, we propose to extend the UCM approach to task functions of the form of Eqs (7) and (8). As expressed in Eq. (7), $\dot{{\bf{e}}}$ is a function of **q** and $\dot{{\bf{q}}}$. The idea is to extend the UCM reasoning by writing down an approximation of $\dot{{\bf{e}}}$ around a particular mean behavior of the participant across trials $(\bar{{\bf{q}}},\bar{\dot{{\bf{q}}}})$, by computing a first order Taylor expansion as in Eq. (2). To this end, the partial derivatives of $\dot{{\bf{e}}}$ need to be computed with respect to **q** and $\dot{{\bf{q}}}$ as follows:

9a $$\frac{\partial \dot{{\bf{e}}}}{\partial \dot{{\bf{q}}}}{|}_{{\bf{q}}=\bar{{\bf{q}}}}\hat{=}A(\bar{{\bf{q}}},\dot{{\bf{q}}}),$$

9b $$\frac{\partial \dot{{\bf{e}}}}{\partial {\bf{q}}}{|}_{\dot{{\bf{q}}}=\bar{\dot{{\bf{q}}}}}\hat{=}B({\bf{q}},\bar{\dot{{\bf{q}}}}).$$

It is straightforward that $A(\bar{{\bf{q}}},\dot{{\bf{q}}})$ does not depend on $\bar{\dot{{\bf{q}}}}$: $A(\bar{{\bf{q}}},\dot{{\bf{q}}})={J}_{e}(\bar{{\bf{q}}})$. The calculation details for *B* are given in Appendix A:

10 $${B}_{ij}=\sum _{k=0}^{n}\frac{\partial {({J}_{e}({\bf{q}}))}_{ik}}{\partial {q}_{j}}{\bar{\dot{q}}}_{k}.$$

This leads to the first order Taylor expansion of $\dot{{\bf{e}}}$ around $(\bar{{\bf{q}}},\bar{\dot{{\bf{q}}}})$:

11 $$\dot{{\bf{e}}}({\bf{q}},\dot{{\bf{q}}})-\dot{{\bf{e}}}(\bar{{\bf{q}}},\bar{\dot{{\bf{q}}}})=\mathop{\underbrace{[\begin{array}{cc}B(\bar{{\bf{q}}},\bar{\dot{{\bf{q}}}})\, & |\,A(\bar{{\bf{q}}},\bar{\dot{{\bf{q}}}})\end{array}]}}\limits_{{J}_{\dot{e}}}[\begin{array}{c}{\bf{q}}-\bar{{\bf{q}}}\\ \dot{{\bf{q}}}-\bar{\dot{{\bf{q}}}}\end{array}]+{\boldsymbol{\varepsilon }}({\bf{q}},\dot{{\bf{q}}}).$$

Similarly, $\ddot{{\bf{e}}}$ is a function of ***q***, $\dot{{\bf{q}}}$ and $\ddot{{\bf{q}}}$ (Eq. (8)). To express its first order Taylor expansion around the mean behavior of the participant ($\bar{{\bf{q}}}$, $\bar{\dot{{\bf{q}}}}$, $\bar{\ddot{{\bf{q}}}}$), the partial derivatives of $\ddot{{\bf{e}}}$ need to be computed with respect to **q**, $\dot{{\bf{q}}}$ and $\ddot{{\bf{q}}}$:

12a $$\frac{\partial \ddot{{\bf{e}}}}{\partial \ddot{{\bf{q}}}}{|}_{\begin{array}{c}{\bf{q}}=\bar{{\bf{q}}}\\ \dot{{\bf{q}}}=\bar{\dot{{\bf{q}}}}\end{array}}\hat{=}C(\bar{{\bf{q}}},\bar{\dot{{\bf{q}}}},\ddot{{\bf{q}}}),$$

12b $$\frac{\partial \ddot{{\bf{e}}}}{\partial \dot{{\bf{q}}}}{|}_{\begin{array}{c}{\bf{q}}=\bar{{\bf{q}}}\\ \ddot{{\bf{q}}}=\bar{\ddot{{\bf{q}}}}\end{array}}\hat{=}D(\bar{{\bf{q}}},\dot{{\bf{q}}},\bar{\ddot{{\bf{q}}}}),$$

12c $$\frac{\partial \ddot{{\bf{e}}}}{\partial {\bf{q}}}{|}_{\begin{array}{c}\dot{{\bf{q}}}=\bar{\dot{{\bf{q}}}}\\ \ddot{{\bf{q}}}=\bar{\ddot{{\bf{q}}}}\end{array}}\hat{=}E({\bf{q}},\bar{\dot{{\bf{q}}}},\bar{\ddot{{\bf{q}}}}).$$

In fact it is straightforward to demonstrate that $C(\bar{{\bf{q}}},\bar{\dot{{\bf{q}}}},\ddot{{\bf{q}}})$ does not depend neither on $\bar{\dot{{\bf{q}}}}$ nor on $\ddot{{\bf{q}}}$: $C(\bar{{\bf{q}}},\bar{\dot{{\bf{q}}}},\ddot{{\bf{q}}})=C(\bar{{\bf{q}}})={J}_{e}(\bar{{\bf{q}}})$. The calculation details for *D* and *E* are given in Appendix A:

13a $${D}_{ij}=\sum _{k=0}^{n}\frac{\partial {({\dot{J}}_{e}(\bar{{\bf{q}}},\dot{{\bf{q}}}))}_{ik}}{\partial {\dot{{\bf{q}}}}_{j}}{\dot{q}}_{k}+{\delta }_{jk}{({\dot{J}}_{e}(\bar{{\bf{q}}},\dot{{\bf{q}}}))}_{ik},$$

13b $${E}_{ij}=\sum _{k=0}^{n}\frac{\partial {({\dot{J}}_{e}({\bf{q}},\bar{\dot{{\bf{q}}}}))}_{ik}}{\partial {q}_{j}}{\bar{\dot{q}}}_{k}+\sum _{k=0}^{n}\frac{\partial {({J}_{e}({\bf{q}}))}_{ik}}{\partial {q}_{j}}{\bar{\ddot{q}}}_{k},$$

with *δ*, the Kronecker delta. This leads to the first order Taylor expansion of $\ddot{{\bf{e}}}$ around $\bar{{\bf{q}}}$, $\bar{\dot{{\bf{q}}}}$ and $\bar{\ddot{{\bf{q}}}}$:

14 $$\begin{array}{rcl}\ddot{{\bf{e}}}({\bf{q}},\dot{{\bf{q}}},\ddot{{\bf{q}}})-\ddot{{\bf{e}}}(\bar{{\bf{q}}},\bar{\dot{{\bf{q}}}},\bar{\ddot{{\bf{q}}}}) & = & \mathop{\underbrace{[E(\bar{{\bf{q}}},\bar{\dot{{\bf{q}}}},\bar{\ddot{{\bf{q}}}})|D(\bar{{\bf{q}}},\bar{\dot{{\bf{q}}}},\bar{\ddot{{\bf{q}}}})|C(\bar{{\bf{q}}})]}}\limits_{{J}_{\ddot{e}}}\\  &  & [\begin{array}{c}{\bf{q}}-\bar{{\bf{q}}}\\ \dot{{\bf{q}}}-\bar{\dot{{\bf{q}}}}\\ \ddot{{\bf{q}}}-\bar{\ddot{{\bf{q}}}}\end{array}]+{\boldsymbol{\varepsilon }}({\bf{q}},\dot{{\bf{q}}},\ddot{{\bf{q}}}\mathrm{).}\end{array}$$

From these matrix formulations of the first order Taylor expansions of $\dot{{\bf{e}}}$ and $\ddot{{\bf{e}}}$ (Eqs (11) and (14)), one can directly apply the UCM approach previously recalled, by computing the null space of ${J}_{\dot{e}}$ and ${J}_{\ddot{e}}$ respectively. Then, by projecting the variation of elemental variables with regard to the mean reference behavior onto the null space and its orthogonal, one can compute **ucm**_*r*_ and **ucm**_⊥,*r*_, as in Eq. (3a,b). The computation of the ITC (Eq. (6)) is then performed to conclude whether the task under study is preferentially controlled by the CNS or not. In the sequel, a direct application of this UCM extension is made to illustrate the framework. To this end, we study linear and angular momenta derivative task functions that are hypothesized to be preferentially controlled by the CNS during jump and landing motions in parkour. Before introducing the experimental procedure and the results, let us show how the proposed extension can be applied to this specific case study.

### An application to linear and angular momenta derivative tasks

The hypothesized tasks are related to the linear and angular momenta derivatives (a justification for this choice is provided in the following section). Momenta tasks can be computed based on the centroidal dynamics: the dynamics expressed at the CoM of the whole body^26^. The centroidal momenta matrix *A*_*G*_(**q**) = *I*_*sys*_*J*_*sys*_, which is the product of the system inertia matrix *I*_*sys*_ and the system Jacobian *J*_*sys*_ as described by Orin *et al*.^27^, maps the system joint velocities $\dot{{\bf{q}}}$ to the centroidal momenta ${{\bf{h}}}_{G}={[{{\bf{p}}}^{T},{ {\mathcal L} }_{G}^{T}]}^{T}$ as follows:

15 $${{\bf{h}}}_{G}={A}_{G}\dot{{\bf{q}}},$$

where ${\bf{p}}={m}_{body}\dot{{\bf{c}}}$ is the linear momentum with *m*_*body*_ the total body mass and $\dot{{\bf{c}}}\in {{\mathbb{R}}}^{3}$ its CoM velocity, and ***L***_*G*_ is the angular momentum of the body expressed at its CoM position ${\bf{c}}\in {{\mathbb{R}}}^{3}$ as:

16 $${ {\mathcal L} }_{G}=\sum _{k=1}^{K}({{\bf{c}}}_{k}-{\bf{c}})\times {{\bf{p}}}_{k}+{ {\mathcal L} }_{k},$$

where ${{\bf{c}}}_{k}\in {{\mathbb{R}}}^{3}$ is the position vector of the CoM of the *k*^*th*^ segment, ${ {\mathcal L} }_{k}$ is the angular momentum of each body segment about its own CoM and ***p***_*k*_ is the linear momentum of the *k*^*th*^ segment.

Furthermore, the time derivative of Eq. (15) gives the momenta derivative and results in the following equation:

17 $${\dot{{\bf{h}}}}_{G}({\bf{q}},\dot{{\bf{q}}},\ddot{{\bf{q}}})={A}_{G}({\bf{q}})\ddot{{\bf{q}}}+{\dot{A}}_{G}({\bf{q}},\dot{{\bf{q}}})\dot{{\bf{q}}},$$

where ${\dot{{\bf{h}}}}_{G}={[{\dot{{\bf{p}}}}^{T},{ {\mathcal L} }_{G}^{T}]}^{T}$ represents the time derivative of the centroidal momenta. This notation is a compact formulation that embeds the three components of the Linear Momentum Derivative (LMD(x), LMD(y) and LMD(z)) and the three components of the Angular Momentum Derivative (AMD(x), AMD(y) and AMD(z)). Eq. (17) matches the pattern of Eq. (8) and thus can be used as a task function under the presented formalism. Hence, the application of the UCM approach to Eq. (17) requires to compute its partial derivatives which are similar to the ones of Eq. (12a–c) and that are developed in Appendix B.

## Materials and Methods

### Precision jump and landing in parkour

In this section, we show how the previously described formalism can be applied to the study of inherently highly dynamic and skilled tasks. For this purpose, we consider a practical case of application to complex parkour motions, referred to as precision jump and landing techniques. First, the parkour practice and the studied techniques are briefly introduced. Afterwards, we present the methodology, results and discussion. Finally, in order to validate our hypothesis with a different approach and compare the obtained results, we also study task variability with the surrogate data approach to high dimensional correlation as proposed by Muller and Sternad^28^. These results are presented and briefly discussed in Appendix C.

**Figure 1:**
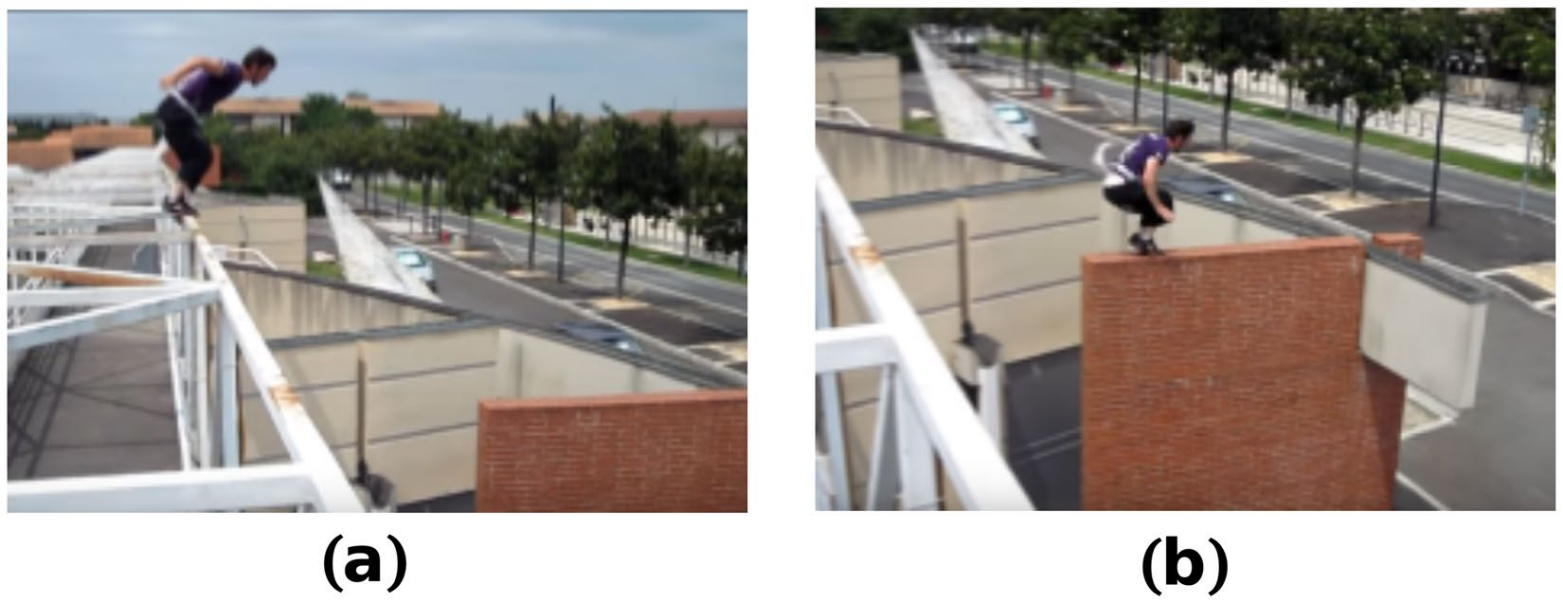
Parkour technique. Parkour precision jump (**a**) and landing (**b**) techniques performed in urban space. The photos were taken at Toulouse (France) and correspond to a parkour expert that was part of the current study.

Parkour techniques are highly skilled and dynamic, and require practitioners–named traceurs–to adapt their movements to the constrained and variable environment to overcome obstacles quickly and efficiently. These motions include versatile jump, landing, climbing and vaulting strategies. In this paper, we focus on precision jump and landing techniques (Fig. 1). These techniques are common to many parkour motions and require highly dynamic motor behaviors to generate and counteract external forces^29–31^. The precision techniques are similar to the standing long jump techniques (horizontal jump performed with both feet simultaneously without run-up) used in athletics. In parkour, they are used for performing relatively small jumps that necessitate precision (Fig. 1). They require practitioners to jump using both feet in parallel while swinging the arms and bending the knees to project the body forwards appropriately. At landing, traceurs have to land with precision on their forefoot without heel contact with the ground, bend their lower-limb joints without any varus-valgus motion of the knees and use their arms to counterbalance the movement and stabilize themselves (Fig. 1).

### Hypothesized task functions

Hypothesized task functions of the parkour technique were chosen according to the literature and the observation of recorded pre-tests. In this context, we expressed motor task functions in terms of linear and angular momenta. Besides testing whether these task functions are preferentially controlled by the CNS or not, our goal was also to check for the existence of a hierarchical structure of functions that could describe the whole motion organization. This hypothesis is motivated by the fact that, in order to execute these motions, the CNS has to organize the control of several motor task functions simultaneously, to guarantee the achievement of the motion goals (e.g. perform a precise landing without tipping over or getting injured).

#### Motion phases

**Figure 2:**
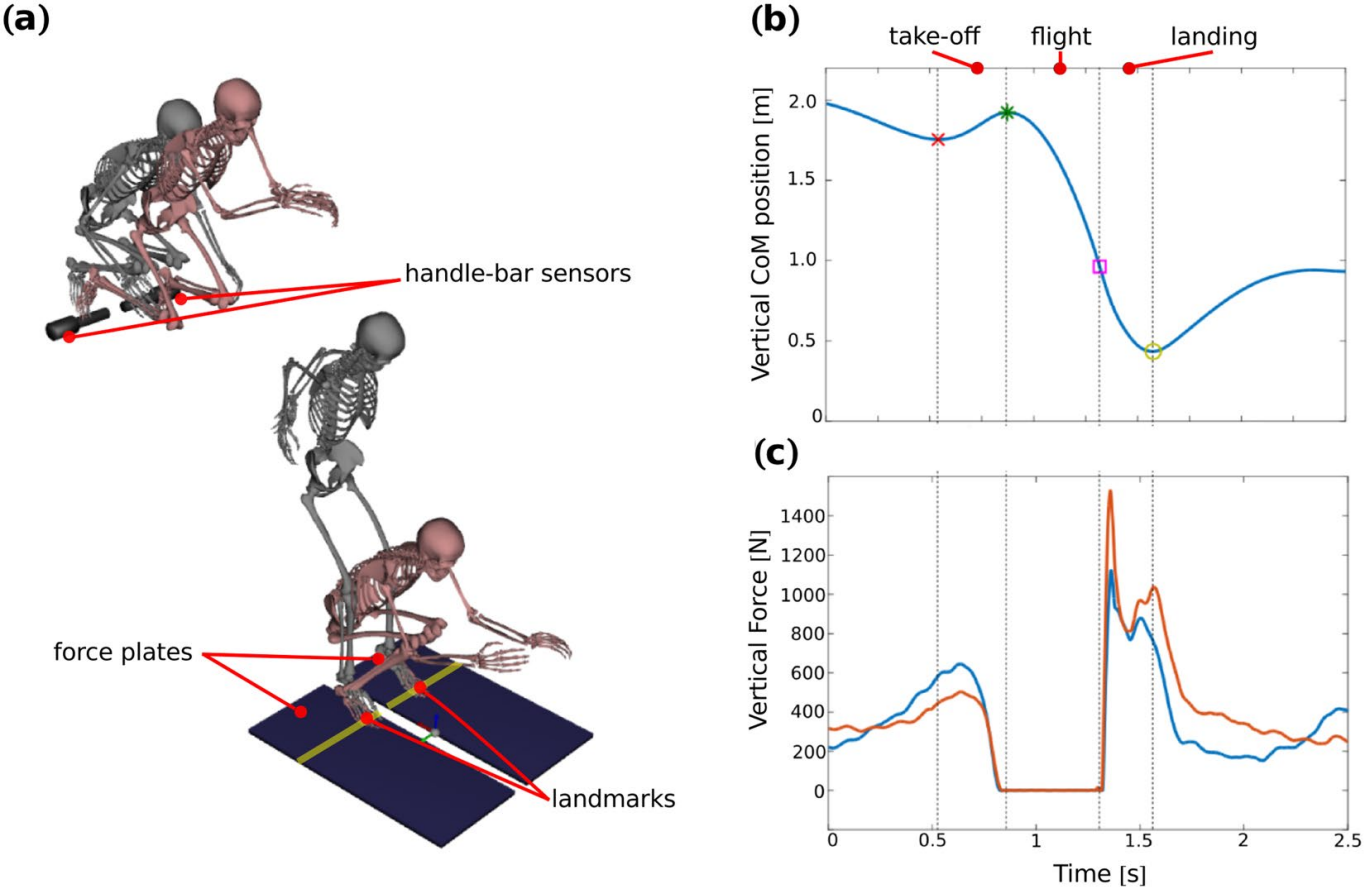
Parkour motion analysis. In (**a**), the first two skeletons illustrate the beginning and the end of the take-off motion while the last two skeletons show the beginning and the end of the landing. In (**b**), the vertical trajectory of the CoM. In (**c**) the vertical reaction force profiles. The CoM trajectory and force profiles correspond to the motion of one participant during the recording sessions and allow for slicing the parkour motion into three sub-motions: take-off, flight and landing.

The motion is divided into three sub-motions: take-off, flight and landing. As our goal was to focus on momenta task functions, which are conserved quantities during the flight (according to Newton’s laws of motion), we decided to exclude the flight motion from our analysis. The take-off phase is defined between the time at which the height of the CoM is minimal and the last foot contact instant. The flight phase was defined between the end of the take-off phase and the initial contact with the ground, defined as the time at which the vertical ground reaction force reaches 50 [N]. The landing phase was defined between initial contact with the ground and the time at which the hight of the CoM becomes minimal. The phases were normalized by time duration from 0% to 100% (Fig. 2).

#### Motor task functions during take-off

Research on the standing long jump motion, which is similar to the parkour motion considered in this study, have shown that during take-off, the impulse profile which modifies the take-off velocity of the CoM constitutes the principal performance factor of this motion^32^. Note that in order to produce the desired impulse profile, the jumper has to generate the necessary forces against the ground which provide the CoM ballistic motion. Furthermore, it has been shown that swinging the arms contributes to increase the impulsion, and therefore the CoM velocity, through joint torque augmentation^33,34^. It has also been demonstrated that swinging the arms improves the performance by alleviating excessive forward rotation^35^ and contributing to position the body segments properly for landing^36^.

Controlling ground reaction forces and swinging the arms during impulse, appear then to be the most relevant strategies for generating an optimal ballistic motion of the CoM. On the other hand, momenta derivatives expressed at the CoM are equal to net external forces and torques according to Newton-Euler equations. Accordingly, we hypothesize that linear and angular momenta derivatives expressed at the CoM should be preferentially controlled by the CNS during the take-off motion through the following task functions:

- “LMD(y,z)”: A task function that reflects the control of the forces to generate the required CoM velocity expressed in terms of the antero-posterior (A-P) and vertical components of the linear momentum derivative “LMD”.
- “AMD(y)”: A task function that enhances the traceur’s performance when jumping and contributes to prepare the body posture for landing in terms of the A-P component of the angular momentum derivative “AMD” expressed at the CoM.

#### Motor task functions during landing

During landing, reducing the vertical ground reaction forces “GRF” (which is the same as reducing the vertical linear momentum derivative) and lowering the loading rate (which is equivalent to control the time to the vertical GRF peak) contribute to prevent pain and injury^37^. It has been shown that traceurs are able to reduce these quantities^31^. Moreover, traceurs are able not only to land safer but also to better control their posture. A study of the center of pressure (CoP) control through the modulation of A-P and medial-lateral (M-L) components of the GRFs provided this evidence^38^. The angular momentum derivative expressed at the CoM position appears also to be regulated during parkour landings^39^. Computing each segment’s contribution to the AMD, revealed that each pair of segments (right/left arms, right/left thighs, etc.) produces two opposed contributions that tend to cancel each other. This observation suggested a 3D whole-body strategy obtained through segmental cancellations. Based on these elements, we hypothesized that the following task functions are preferentially controlled by the CNS during landing:

- “LMD(z)”: A task function that reflects a strategy to guarantee a safe landing execution by lowering the vertical reaction forces and the loading rate. This task function is expressed in terms of the vertical component of the LMD.
- “LMD(x,y)”: A task function that reflects a strategy of postural control in terms of the M-L and A-P components of the LMD.
- “AMD(x,y,z)”: A task function that reflects a falling-down avoidance strategy through rotational motions around the body principal axes expressed in terms of the AMD. This task function might also contribute to injury prevention by decreasing the varus-valgus motion of the knees.

Before contacting with the ground, participants pre-activate their muscles in preparation for landing^40^ and the level of excitability of stretch reflexes are likely to change before initial contact with the ground^41^. Thus, landing was analyzed from 4% of the motion to ensure consistency of our interpretations.

### Participants

Seven healthy trained male traceurs (age: 23.5 ± 3.1 y, height: 1.72 ± 0.07 m, mass: 69.2 ± 6.8 kg) volunteered for this study. Their experience in parkour practice was 5.7 ± 2.8 y. The subject exclusion criterion was based on history of lower extremity injuries or diseases that might affect landing biomechanics. Prior to participation in the experiments, informed written consent was obtained from participants. The experiments were conducted in accordance with the standards of the Declaration of Helsinki (rev. 2013) and approved by the ethics evaluation committee of INSERM (IORG0003254,FWA00005831) and the Institutional Review Board (IRB00003888) of the French Institute of medical research and Health.

### Experimental protocol

Participants performed a warming up session followed by a familiarization period during which they were provided with the protocol instructions and they got accustomed to the laboratory environment. A total of 8 successful repetitions per participant was recorded according to our pre-tests during which we verified that fatigue and pain were minimized. During the experimental protocol, fatigue was controlled by including resting times of at least 3 minutes between repetitions^31^ and by getting oral feedback from practitioners. The landing protocol included a jump from 75% of the participant height toward a target landmark placed on the floor at a distance equal to the square of the jump height (Fig. 2). Participants were asked to land on the target landmark and to stabilize their posture by using the parkour precision technique. Only successful trials were taken into account.

### Skeletal model

**Figure 3:**
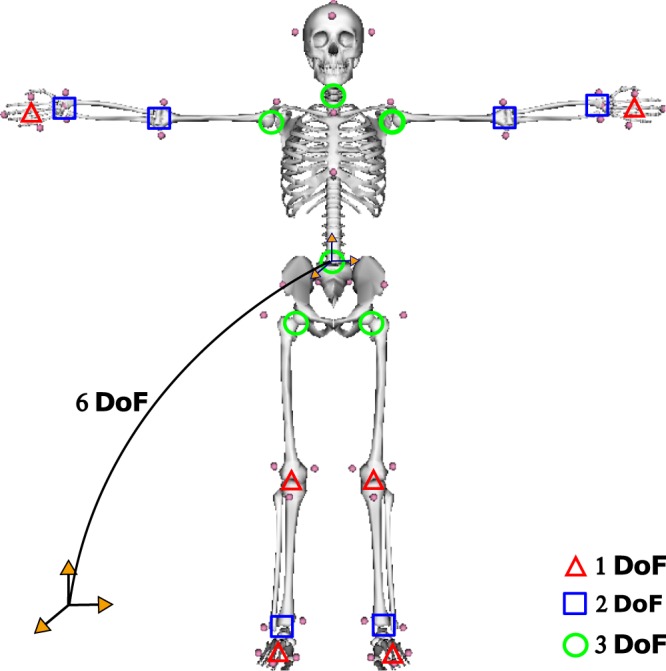
Modeling of the human skeletal system. Whole-body skeletal model of the human body used to study the parkour mouvements.

A whole-body 3D model including 42 degrees of freedom and 19 body segments was used to reconstruct the main elementary movements of the traceur (Fig. 3). This model is limited to joints that are relevant for the task and includes some simplifications. A 3D model is required because, as shown in previous studies^42^, a simpler sagittal model is not sufficient to study the upper body motion during standing long jumps.

The characteristics of the model are listed below:

- The lower limb, pelvis and upper limb anthropometry are based on the running model of Hamner *et al*.^43^. Mass properties of the torso and head segments (the head and neck are modeled as one segment) are estimated from the regression equations of Dumas *et al*.^44^. The anthropomorphic description of the hands is based on the regression equations of De Leva *et al*.^45^.
- Each lower extremity has seven degrees of freedom. The hip is modeled as a ball-and-socket joint, the knee is modeled as a revolute joint, the ankle is modeled as 2 revolute joints (flexion-extension and inversion-eversion), and the toes are modeled with one revolute joint at the metatarsals.
- The pelvis joint is modeled as a free-flyer joint to permit the model to translate and rotate in the 3D space, the lumbar motion is modeled as a ball-and-socket joint^46^ and the neck joint is also modeled as a ball-and-socket joint.
- Each arm is modeled with 8 degrees of freedom. The shoulder is modeled as a ball-and-socket joint, the elbow and forearm rotations are modeled with revolute joints to represent flexion-extension and pronation-supination^47^, the wrist flexion-extension and radial-ulnar deviations are modeled with revolute joints, and the hand fingers are modeled with one revolute joint for all fingers.
- The model includes a whole-body marker set with 48 markers placed on anatomical landmarks as suggested by Wu *et al*.^48,49^. The visual elements are based on the running model of Hamner *et al*.^43^.

### Data acquisition

Whole-body 3D kinematic data were collected by 14 infra-red cameras sampling at 400 Hz (Vicon, Oxford Metrics, Oxford, UK) that recorded the motion of 48 reflective markers placed on the participant body according to Wu *et al*.^48,49^ and Dumas *et al*.^44^ recommendations as follows: the first, and fifth metatarsals, second toe tip, calcaneus, lateral and internal malleolus, anterior tibial tuberosity, medial and lateral epicondyles of knee, greater trochanter, posterior superior iliac spine and anterior superior iliac spine, *procesuss xiphoideus*, *incisura jugularis*, seventh cervicale, tenth thoracic vertebra, acromioclavicular, medial and lateral epicondyle, ulnar and radial *styloid*, second and fifth metacarpal heads, second fingertip, *sellion*, *occiput*, right and left temporal (Fig. 3). Two force plates (AMTI, Watertown, MA, USA) embedded into the floor and two rigid handle bar sensors (∅ 63 mm, SENSIX, Poitiers,Vienne, France) placed on a parkour tubular structure were used to record external forces at 2 kHz (Fig. 2). Force data were used to double-check the computations of the momenta derivative and to define the onsets of the motion phases (% of the motion).

### Data analysis

Kinematics and kinetics were processed with the same cut-off frequency^50^ using a low-pass Butterworth digital filter of 4th order applied in zero-phase. A cut-off frequency of 35 Hz was used after a residual analysis^51^. The introduced whole-body model was used to compute inverse kinematics which was solved with OpenSim^52^ by minimizing the squared distance between recorded and virtual markers^53^ and by using the Euler xyz body-fixed rotation angles^54^. Computations of AMD and ITC were performed using a custom-made program and a physics engine^55^. The free-flyer joint (root frame, six degrees of freedom) was not taken into account in the control variables. Indeed, according to the dynamic equations of poly-articulated systems, this joint is under-actuated by the action of the body joints and therefore cannot be directly controlled.

### Statistical analysis

Individual statistical studies were conducted for the take-off and landing motions. All data are presented as the mean ± the confidence interval. The normality of the data was assessed with the Shapiro–Wilk test. The *p*-value for determining statistical significance of hypotheses was *p* = 0.05. To statistically verify if motor task functions were preferentially controlled by the CNS, unpaired *t*-tests comparing the ITC value with a zero mean were performed. To investigate if task hierarchies can be identified in the organization of the motion and to assess the temporal evolution of ITC values during each motion, a repeated measures ANOVA (task × phase, takeoff: 2 × 4, landing: 3 × 5) with the phases and the tasks as within-subjects factors was performed. Paired *t*-tests with the Bonferroni correction were then carried out to assess main effects. Finally, the eta-squared method was used to test the effect size on the measures and the power of the statistics. All statistics were computed using R^56^.

## Results

The Index of Task Control (ITC) refers to the ratio between the variance in the UCM and in its orthogonal. According to the UCM hypothesis, when the ITC is greater than 0, the motor task is considered as being preferentially controlled by the CNS. ITC values were always positive for all the hypothesized task functions during take-off and landing. Moreover, task functions had significantly different levels of ITC.

### Take-off

**Figure 4:**
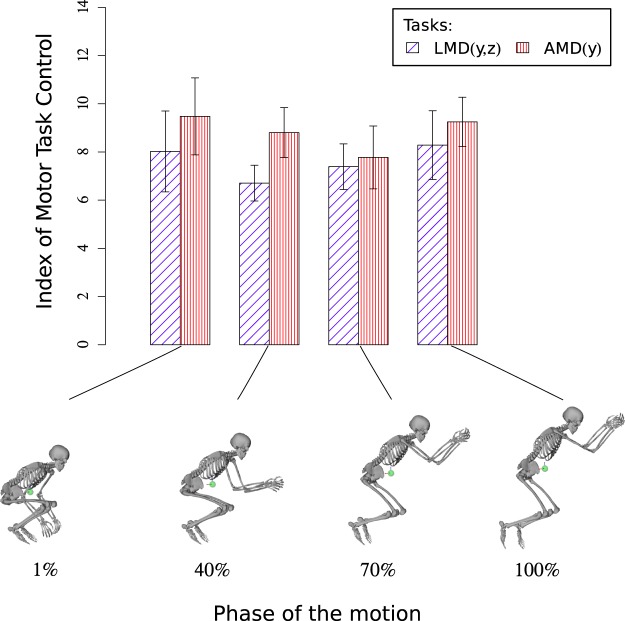
ITC values at take-off. On bottom: snapshots of the reconstructed motion taken at each phase of the motion (1, 40, 70 and 100%). On top: the corresponding mean (±confidence intervals) values of the indexes of motor task control (ITC) during the take-off motion for the LMD(y,z) and the AMD(y) task functions.

Each of the two hypothesized task functions turned out to be preferentially controlled at each motion phase ($p < 0.001$) (Table 1). Indeed, during the whole motion, ITC indexes are higher than 0. Thus, the derivative of the linear momentum along y and z (LMD(y,z)) and the derivative of the angular momentum around z (AMD(y)) appear to be preferentially controlled by the CNS during the take-off (Fig. 4). A repeated measures analysis of variance of the ITC emphasized a main effect for the task factor ($F(1,6)=36.77,\,p < 0.001,\,{\eta }^{2}=0.86,\,f=2.47$). No significant effect was observed for the phase factor ($F(3,18)=2.74,\,p=0.071,\,{\eta }^{2}=0.31,\,f=2.47$). Furthermore, post-hoc comparisons using paired t-tests with the Bonferroni correction (*p*/4) indicated that task functions ITCs were significantly different at the 1% (*p* = 0.005), at 40% (*p* = 0.002) and at 100% of the motion (*p* = 0.042).

**Table 1:** Hierarchical organization of the take-off motion in terms of the task functions.

	Phases			
	1%	40%	70%	100%
1	AMD(y)*	AMD(y)*	AMD(y)	AMD(y)*
2	LMD(y,z)	LMD(y,z)	LMD(y,z)	LMD(y,z)

*Significantly different from LMD(y,z).

### Landing

**Figure 5:**
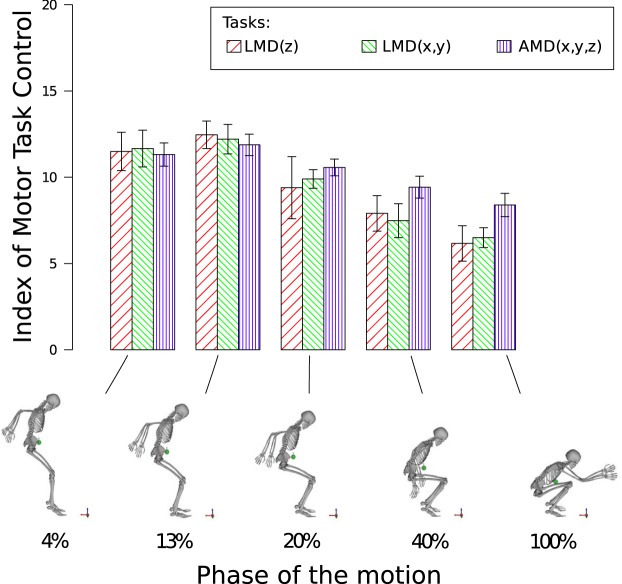
ITC values at landing. On bottom: snapshots of the reconstructed motion taken at each phase of the motion (4, 13, 20, 40 and 100%). On top: the corresponding mean (±confidence intervals) values of the indexes of motor task control (ITC) during the landing motion for the LMD(z), the LMD(x,y) and the AMD(x,y,z) task functions.

Both the derivatives of the angular and linear momenta in the three directions were preferentially controlled by the CNS at each motion phase ($p < 0.001$) during landing (Fig. 5). A repeated measures analysis of variance of the ITC indicated a main effect for the task factor ($F(2,12)=\mathrm{14.23,}\,p < \mathrm{0.001,}\,{\eta }^{2}=0.70,\,f=1.54$) and for the phase factor ($F(4,24)=67.4,p < 0.001,{\eta }^{2}=0.91,f=3.35$). Post-hoc comparisons using paired t-tests with Bonferroni correction corroborated that task functions (*p*/15) were significantly different ($p < 0.001$) (Table 2) and that ITC values of the phases (*p*/10) evolved differently with time (Table 3). Figure 5, displays the decrease of each task function after the first 13% of the motion. This shows that the control appears to be more important at the beginning of the landing phase, for the LMDs and AMD task functions. Moreover, Table 2 shows that at 20%, 40% and 100% of the landing phase, AMD(x,y,z) and LMD(x,y)’s ITCs were significantly different and that at 13%, 40% and 100%, AMD(x,y,z) and LMD(z)‘s ITCs were significantly different. Table 3 contains the evolution of each ITC index during the landing phase. LMD(z)‘s ITC was significantly smaller at 40% and 100% with regard to 4% and 13% (it decreased at the end of the landing phase). In a same manner, the ITC values of LMD(x,y) and AMD(x,y,z) significantly decreased during the landing phase. Table 4 shows which task function was prioritized by the CNS at each phase of the landing: LMD(z) at 13% of the landing phase, then AMD(x,y,z) from 20%, up to 100%).

**Table 2:** Pairwise comparisons using paired t-tests for the task factor in the landing phases.

Phases					
Task Functions Comparison	4%	13%	20%	40%	100%
LMD(z) & LMD(x,y)	1	1	1	1	1
LMD(x,y) & AMD(x,y,z)	0.94	0.59	0.044	0.003	<0.001
LMD(z) & AMD(x,y,z)	1	0.011	0.275	0.005	0.004

*p*–values were adjusted with the Bonferroni correction.

**Table 3:** Pairwise comparisons using paired t-tests for the phase factor in the landing phases.

LMD(z)					LMD(x,y)				AMD(x,y,z)			
Phase	4%	13%	20%	40%	4%	13%	20%	40%	4%	13%	20%	40%
13%	0.674	—	—	—	1	—	—	—	1	—	—	—
20%	0.284	0.092	—	—	0.102	0.005	—	—	1	0.05	—	—
40%	0.007	0.001	0.592	—	0.008	<0.001	0.005	—	0.064	0.002	0.065	—
100%	0.002	<0.001	0.079	0.088	<0.001	<0.001	<0.001	0.041	0.005	<0.001	0.024	0.067

*p*-values were adjusted with the Bonferroni correction.

**Table 4:** Hierarchical organization of the landing motion in terms of the task functions.

	Phases				
	4%	13%	20%	40%	100%
1	LMD(x,y)	LMD(z)^†^	AMD(x,y,z)*	AMD(x,y,z)*,^‡^	AMD(x,y,z)*,^‡^
2	LMD(z)	LMD(x,y)	LMD(x,y)	LMD(z)	LMD(x,y)
3	AMD(x,y,z)	AMD(x,y,z)	LMD(z)	LMD(x,y)	LMD(z)

*Significantly different from LMD(x,y); ^†^Significantly different from AMD(x,y,z); ^‡^Significantly different from LMD(z).

## Discussion

The hypothesized motor task functions in this study were chosen according to the physics of the motion and the literature. The UCM extension was used to verify whether these task functions are being preferentially controlled by the CNS during the parkour motion. Results reveal complex whole-body motion strategies which lead to the control of the hypothesized dynamic task functions. The higher the ITC, the more the regulation of the task function appears to be preferentially controlled by the CNS. As a consequence, the values of ITCs associated to different task functions reveal the level of involvement of each of them in the achievement of the task. This involvement is interpreted in terms of task hierarchy in the proposed framework. Moreover, the time decomposition of the motion phases reveals that the amount of control dedicated to a task function might not depend on the task function only but also on the phase of the motion. This might be interpreted as a strategy of the CNS to organize motor control in terms of temporal and inter-tasks priorities. Thus, the ITC values provide information about the temporal evolution of task function hierarchies during the motion. In the sequel, we discuss the results obtained by analyzing the proposed extension of the UCM approach to take-off and landing motions in parkour.

### Take-off

The ITC of the LMD(y,z) task function, which is expressed in terms of the A-P and vertical components of the linear momentum derivative, reveals that this task function is preferentially controlled during the take-off motion. This is consistent with the physics of the problem: the traceur has to induce a certain velocity to his CoM in order to precisely determine the ballistic motion that the body will be subject to during the flight phase^32^. This velocity is obtained by integrating over time the acceleration of the CoM, which is the result of the forces applied by the participant on the ground (Newton’s second law). The time integration of the forces exerted on the ground must therefore be precisely controlled at each time step during the take-off.

The AMD(y) task function, which is expressed in terms of the A-P component of the angular momentum derivative expressed at the CoM, is also preferentially controlled by the CNS during the take-off phase. Its ITC value turns out to be greater than in the LMD(y,z) task function throughout the take-off motion. This result makes sense with regard to Euler’s law of motion and the conservation of angular momentum in the sense that, after the very moment of contact loss and during the whole flight, traceurs will not be able to change their angular momentum. As a consequence, excessive rotational motions must be avoided. Another reason for traceurs to control the AMD(y) before flying is to ensure they can reach an appropriate posture in preparation for landing^36^. The leaning forward position of the traceur (Fig. 4) and the gravity force, are used to generate a net torque at the CoM which has to be counteracted throughout the take-off in order to reach the desired angular momentum. To this end, the observed strategy of the arms, pelvis, trunk and head swings contributes to counterbalance the forward sagittal angular momentum^35^. This upper body strategy permits lower limbs to be more involved in the production of the impulsion torques without having to compensate for the forward momentum^35^. This complex inter-limb coordination highlights the importance of controlling the AMD(y) task function as corroborated by our results.

The AMD(y) task function is significantly more controlled at the beginning and at the end of the take-off. This might reflect that ensuring a correct body posture before landing is more important than generating the desired ballistic trajectory of the CoM. A wrong control of the angular momentum during the take-off might imply falling down and getting injured at landing.

### Landing

The LMD(z) task function, which is expressed in terms of the vertical component of the linear momentum derivative, is controlled throughout the landing motion. From the beginning and up to 20% of the motion, the vertical force is controlled so that the peak loading rate and the peak forces can be reduced^31^. This contributes to prevent pain and injuries^37^. During the remaining of the landing, this task function is kept controlled. This is not surprising when considering that the time integration of this force provides the vertical deceleration of the CoM.

The LMD(x,y) task function, which is expressed in terms of the M-L and A-P components of the linear momentum derivative, is also controlled during the whole landing. This is expected during this process because the resulting GRF direction, which depends on the amount of M-L and A-P forces with regard to the normal force must remain inside the friction cones of contact in order to avoid slipping. Moreover, the LMD(x,y) task function affects the center of pressure (CoP) position, which reflects the neuromuscular postural control. Note that, in order to reach static equilibrium–which is the final goal of the parkour technique–, the CoP must finally coincide with the projection of the CoM on the ground. The LMD(x,y) task function is also linked to the forward deceleration of the CoM.

The AMD(x,y,z) task function, which is expressed in terms of the 3 components of the angular momentum derivative expressed at the CoM, is also controlled during landing. Due to the conservation of angular momentum principle, the value of the AMD before the impact is zero. Right after it, because of external forces and torques, the AMD increases. One way to compensate this augmentation in order to keep balance, is to limit the derivative of joint torques during the landing phase. One can note that the pre-activation of muscles in preparation for landing^40^ contributes to keep the derivative of joint torques into reasonable bounds. This strategy might be also responsible for the apparent smoothness of the motion in terms of compliance and style. Furthermore, controlling the AMD(x,y,z) around the vertical axis contributes to reduce the varus-valgus motion and can be an injury prevention mechanism.

Two hierarchies of task functions and a temporal evolution of the ITC value were observed during the landing phase. At 13% of the motion, traceurs appear to control more the LMD(z) task function. The AMD(x,y,z) task function is significantly more controlled throughout the end of landing (from 20% to 100%). Note that after 20% of the landing, the vertical force is lowered. This control might be used to perform small postural adjustments thanks to the observed segment cancellation strategy^39^.

## Conclusion

This study proposed a general framework for identifying dynamic task functions that appear to be preferentially controlled by the CNS in humans, based on the task function approach used in robotics and on an extension of the UCM approach. This theoretical contribution is illustrated by the study of parkour movements for which task function are formulated in terms of whole-body linear and angular momenta derivatives. Based on the analysis of the considered parkour motions, reasonable assumptions about which physical quantities are preferentially controlled by the CNS during the take-off and landing phases were made. By using the proposed UCM extension to study the organization of highly dynamic and skilled tasks, consistent computational arguments were provided to demonstrate that different components of linear and angular momenta are organized by the CNS which exploits motor abundance during successive phases of the parkour motion. This study also reveals the potential of our method for identifying the hierarchical organization of task functions during complex human motions. This organization should be useful by the CNS to generate precise, dynamic, stable and injury-free motions.

The large ITC values of the task functions during take-off and the landing phase might reflect in part the fact that highly skilled and dynamic motions require large amounts of coordination and hence large covariation effects of the elemental variables for stabilizing the linear and angular momenta task functions. Moreover, note that in our study we did not consider the flight phase. In fact, if the same analysis had been performed for this phase, we could have observed some covariance related to the biomechanics of the movement rather than to the control strategy of the CNS. This is true because the angular momentum is conserved and the linear momentum is only affected by gravity, when no other external forces are acting on the body or when these forces can be neglected (e.g. air resistance force during the flight phase of the parkour jump). Note also that, unlike under pure kinematic hypotheses, it is not possible when dealing with task functions expressed in terms of the centroidal dynamics (e.g. LMD and AMD in our work) to ignore segments that downstream from the segment of interest in the kinematic chain. What would be possible is to study the covariation structure of sub-chains linked to the CoM.

The proposed extension of the existing approach to the study of human motions where the dynamics matter is also promising for the synthesis of task-based motion generation algorithms for anthropomorphic systems. These results might also be useful in the frameworks of computer animation and humanoid robotics to generate human-inspired motions. Future works might be oriented towards the testing of different tasks under other experimental protocols to demonstrate the sensitivity of the proposed method with non-controlled task functions. The main limitation of the proposed approach lies in the inherent errors of biomechanical methods that are related to human movement analysis (experimental protocol, scaling of anthropomorphic data, inverse kinematics,…).

## Electronic supplementary material

Appendices A, B and C

## Data Availability

The datasets generated and analyzed during the current study are available from the corresponding author upon request.
