# Supplementary material for: On the coordination of highly dynamic human movements: an extension of the Uncontrolled Manifold approach applied to precision jump in parkour
Source: Sci Rep. 2018 Aug 15;8:12219. doi: 10.1038/s41598-018-30681-6 (PMC6093881; doi:10.1038/s41598-018-30681-6)
Supplement: Supplementary file 1 — Appendices A, B and C [file 41598_2018_30681_MOESM1_ESM.pdf]

On the coordination of highly dynamic human movements: an extension of the Uncontrolled Manifold approach applied to precision jump in parkour.

Galo Maldonado, François Bailly, Philippe Souères, Bruno Watier.

Appendices A, B and C

A Calculation details of the UCM extension applied to kinematic and dynamic task functions

Partial derivatives of $\dot{e}$ with respect to q and $\dot{q}$

$$B(\boldsymbol{q},\bar{\dot{\boldsymbol{q}}}) = \frac{\partial J(\boldsymbol{q})\bar{\dot{\boldsymbol{q}}}}{\partial \boldsymbol{q}}.$$

Let us write $J(\boldsymbol{q})\bar{\dot{\boldsymbol{q}}}$ component-wise:

$$(J(\boldsymbol{q})\bar{\dot{\boldsymbol{q}}})_i = \sum_{k=0}^{n} (J(\boldsymbol{q}))_{ik}\bar{\dot{q}}_k.$$

This leads to the component-wise expression of B:

$$B_{ij} = \frac{\partial (J(\boldsymbol{q})\bar{\dot{\boldsymbol{q}}})_i}{\partial q_j} = \sum_{k=0}^{n} \frac{\partial (J(\boldsymbol{q}))_{ik}}{\partial q_j}\bar{\dot{q}}_k.$$

Note that configuration $\boldsymbol{q}$ contains rotational joints which are elements of the special orthogonal group $SO(3)$ and have to be time differentiated accordingly.

Partial derivatives of $\ddot{e}$ with respect to q, $\dot{q}$ and $\ddot{q}$

Calculation of D

$$D(\bar{\boldsymbol{q}},\dot{\boldsymbol{q}},\bar{\dot{\boldsymbol{q}}}) = \frac{\partial (\dot{J}(\bar{\boldsymbol{q}},\dot{\boldsymbol{q}})\dot{\boldsymbol{q}})}{\partial \dot{\boldsymbol{q}}}.$$

Let us write $\dot{J}(\bar{\boldsymbol{q}},\dot{\boldsymbol{q}})\dot{\boldsymbol{q}}$ component-wise:

$$(\dot{J}(\bar{\boldsymbol{q}},\dot{\boldsymbol{q}})\dot{\boldsymbol{q}})_i = \sum_{k=0}^{n} (\dot{J}(\bar{\boldsymbol{q}},\dot{\boldsymbol{q}}))_{ik}\dot{q}_k.$$

This leads to the component-wise expression of D:

$$D_{ij} = \frac{\partial (\dot{J}(\bar{\boldsymbol{q}},\dot{\boldsymbol{q}})\dot{\boldsymbol{q}})_i}{\partial \dot{\boldsymbol{q}}_j},$$

$$D_{i,j} = \sum_{k=0}^{n} \frac{\partial ((\dot{J}(\bar{\boldsymbol{q}},\dot{\boldsymbol{q}}))_{ik}\dot{q}_k)}{\partial \dot{\boldsymbol{q}}_j},$$

$$D_{i,j} = \sum_{k=0}^{n} \frac{\partial (\dot{J}(\bar{\boldsymbol{q}},\dot{\boldsymbol{q}}))_{ik}}{\partial \dot{\boldsymbol{q}}_j}\dot{q}_k + \delta_{jk}(\dot{J}(\bar{\boldsymbol{q}},\dot{\boldsymbol{q}}))_{ik},$$

with $\boldsymbol{\delta}$ the Kronecker delta.

Calculation of E

$$E(\boldsymbol{q},\bar{\dot{\boldsymbol{q}}},\bar{\ddot{\boldsymbol{q}}}) = \frac{\partial (\dot{J}(\boldsymbol{q},\bar{\dot{\boldsymbol{q}}})\bar{\dot{\boldsymbol{q}}})}{\partial \boldsymbol{q}} + \frac{\partial (J(\boldsymbol{q})\bar{\ddot{\boldsymbol{q}}})}{\partial \boldsymbol{q}}.$$

Let us write $\dot{J}(\boldsymbol{q},\bar{\dot{\boldsymbol{q}}})\bar{\dot{\boldsymbol{q}}}$ component-wise:

$$(\dot{J}(\boldsymbol{q},\bar{\dot{\boldsymbol{q}}})\bar{\dot{\boldsymbol{q}}})_i = \sum_{k=0}^{n} (\dot{J}(\boldsymbol{q},\bar{\dot{\boldsymbol{q}}}))_{ik}\bar{\dot{q}}_k.$$

Let us write $J(\mathbf{q})\bar{\ddot{\mathbf{q}}}$ component-wise:

$$(J(\boldsymbol{q})\bar{\ddot{\boldsymbol{q}}})_i = \sum_{k=0}^{n} (J(\boldsymbol{q}))_{ik}\bar{\ddot{q}}_k.$$

This leads to the component-wise expression of E:

$$E_{ij} = \frac{\partial(\dot{J}(\boldsymbol{q},\bar{\dot{\boldsymbol{q}}})\bar{\dot{\boldsymbol{q}}})_i}{\partial q_j} + \frac{\partial(J(\boldsymbol{q})\bar{\ddot{\boldsymbol{q}}})_i}{\partial q_j},$$

$$E_{ij} = \sum_{k=0}^{n} \frac{\partial(\dot{J}(\boldsymbol{q},\bar{\dot{\boldsymbol{q}}}))_{ik}}{\partial q_j}\bar{\dot{q}}_k + \sum_{k=0}^{n} \frac{\partial(J(\boldsymbol{q}))_{ik}}{\partial q_j}\bar{\ddot{q}}_k.$$

B Application of the UCM extension to the derivative of the centroidal momenta task functions

The similarity between Eq. (8) and Eq. (17) provides a direct case of application of our extension of the UCM theory. To this end, we compute the partial derivatives of $\dot{\boldsymbol{h}}_G(\boldsymbol{q},\dot{\boldsymbol{q}},\ddot{\boldsymbol{q}})$ around the mean performance of one participant ($\bar{\boldsymbol{q}}$, $\bar{\dot{\boldsymbol{q}}}$, $\bar{\ddot{\boldsymbol{q}}}$):

$$\left.\frac{\partial \dot{\boldsymbol{h}}_G}{\partial \ddot{\boldsymbol{q}}}\right|_{\substack{\boldsymbol{q}=\bar{\boldsymbol{q}}\\ \dot{\boldsymbol{q}}=\bar{\dot{\boldsymbol{q}}}}} = A_G(\dot{\boldsymbol{q}}), \tag{1a}$$

$$\left.\frac{\partial \dot{\boldsymbol{h}}_G}{\partial \dot{\boldsymbol{q}}}\right|_{\substack{\boldsymbol{q}=\bar{\boldsymbol{q}}\\ \ddot{\boldsymbol{q}}=\bar{\ddot{\boldsymbol{q}}}}} = \frac{\partial(\dot{A}_G(\bar{\boldsymbol{q}},\dot{\boldsymbol{q}})\dot{\boldsymbol{q}})}{\partial \dot{\boldsymbol{q}}}, \tag{1b}$$

$$\left.\frac{\partial \dot{\boldsymbol{h}}_G}{\partial \boldsymbol{q}}\right|_{\substack{\dot{\boldsymbol{q}}=\bar{\dot{\boldsymbol{q}}}\\ \ddot{\boldsymbol{q}}=\bar{\ddot{\boldsymbol{q}}}}} = \frac{\partial(\dot{A}_G(\boldsymbol{q},\bar{\dot{\boldsymbol{q}}})\bar{\dot{\boldsymbol{q}}})}{\partial \boldsymbol{q}} + \frac{\partial(A_G(\boldsymbol{q})\bar{\ddot{\boldsymbol{q}}})}{\partial \boldsymbol{q}}. \tag{1c}$$

Then we apply the presented framework to the first order Taylor expansion of $\dot{\boldsymbol{h}}_G$.

C Applying the Covariation by Randomization method to our dataset

The Covariation by Randomization (CR) method proposed by[1] is intended to be used for multidimensional systems with strong non-linearities where analysis of variance is made using a common metric in the task space. This approach compares the variance of task related variables obtained with the recorded human data to the variance of these variables generated by surrogate data. Surrogate data is obtained by randomly permuting elemental variables with repetitions in order to get rid-off possible correlations between these variables. Although this method looks suitable for dealing with our data set, it fails to interpret variance as a measure of stability. Moreover, structure of variance that is not caused by correlation but by different amounts of variance in the elemental variables is undetected in this approach. See[3] for a critical comparison between the UCM and CR approaches. However, the results that come out of this analysis corroborate the fact that the results obtained with the UCM method are not the consequence of artifacts from highly covarying elemental variables, that would inherently not contribute to the task. In this appendix we provide the results of the CR approach applied to our data set. An index of covariation (ICR) was calculated by obtaining surrogate data with 9 permutations (Figure 1 and Figure 2 of this appendix). Note that the same log transformation was applied to the index of covariation in accordance with our ITC calculations.

All results suggest correlation between elemental variables (IC > 0). Note that IC and ITC values does not have to be the same (see[2] for a detailed explication). Finally, note that the task function formalism presented in this manuscript also generalizes with the CR approach.

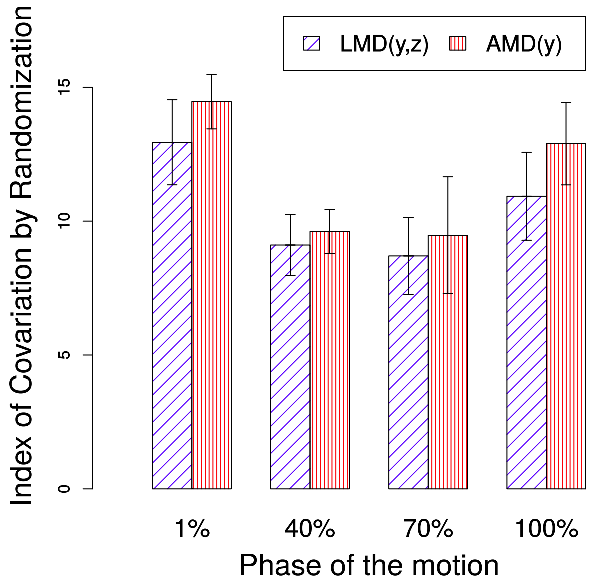

Figure 1. ICR values at take-off. Mean (± confidence intervals) values of the indexes of covariation by randomization (CR) during the take-off motion for the LMD(y,z) and the AMD(y) task.

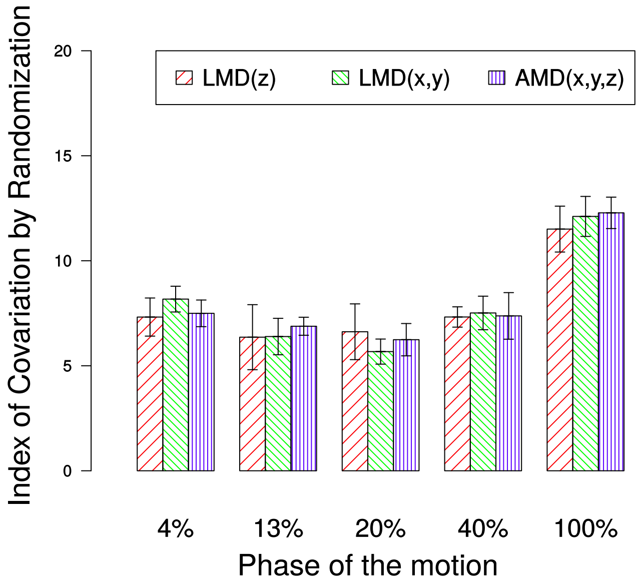

Figure 2. ICR values at landing. Mean (± confidence intervals) values of the indexes of covariation by randomization (CR) during the landing motion for the LMD(z), the LMD(x,y) and the AMD(x,y,z) task.

References

1. Hermann Müller and Dagmar Sternad. A randomization method for the calculation of covariation in multiple nonlinear relations: illustrated with the example of goal-directed movements. Biological cybernetics, 89(1):22–33, 2003.
2. Julius Verrel. A formal and data-based comparison of measures of motor-equivalent covariation. Journal of neuroscience methods, 200(2):199–206, 2011.
3. Gregor Schöner and John P Scholz. Analyzing variance in multi-degree-of-freedom

movements: uncovering structure versus extracting correlations. Motor control, 11(3):259–275, 2007.
